# Identification of mutations in *HEXA* and *HEXB* in Sandhoff and Tay-Sachs diseases: a new large deletion caused by *Alu* elements in *HEXA*

**DOI:** 10.1038/hgv.2018.3

**Published:** 2018-03-15

**Authors:** Hassan Dastsooz, Mohsen Alipour, Sanaz Mohammadi, Fatemeh Kamgarpour, Fatemeh Dehghanian, Majid Fardaei

**Affiliations:** 1 Comprehensive Medical Genetic Center, Shiraz University of Medical Sciences, Shiraz, Iran; 2 Medical Genetics Department, Shiraz University of Medical Sciences, Shiraz, Iran

## Abstract

G_M2_ gangliosides are a group of lysosomal lipid storage disorders that are due to mutations in *HEXA*, *HEXB* and *GM2A*. In our study, 10 patients with these diseases were enrolled, and Sanger sequencing was performed for the *HEXA* and *HEXB* genes. The results revealed one known splice site mutation (c.346+1G>A, IVS2+1G>A) and three novel mutations (a large deletion involving exons 6–10; one nucleotide deletion, c.622delG [p.D208Ifsx15]; and a missense mutation, c.919G>A [p.E307K]) in *HEXA*. In *HEXB*, one known mutation (c.1597C>T [p.R533C]) and one variant of uncertain significance (c.619A>G [p.I207V]) were identified. Five patients had c.1597C>T in *HEXB*, indicating a common mutation in south Iran. In this study, a unique large deletion in *HEXA* was identified as a homozygous state. To predict the cause of the large deletion in *HEXA*, RepeatMasker was used to investigate the *Alu* elements. In addition, to identify the breakpoint of this deletion, PCR was performed around these elements. Using Repeat masker, different *Alu* elements were identified across *HEXA*, mainly in intron 5 and intron 10 adjacent to the deleted exons. PCR around the *Alu* elements and Sanger sequencing revealed the start point of a large deletion in *Alu*Sz6 in the intron 6 and the end of its breakpoint 73 nucleotides downstream of *Alu*Jo in intron 10. Our study showed that *HEXA* is an *Alu*-rich gene that predisposes individuals to disease-associated large deletions due to these elements.

## Introduction

GM2 gangliosides are a group of lysosomal lipid storage disorders that resulted from disease-causing mutations in three autosomal recessive genes, including hexosaminidase A (*HEXA*), hexosaminidase subunit beta (*HEXB*) and GM2 ganglioside activator (*GM2A*). The *HEXB* gene produces a subunit of two related enzymes that include beta-hexosaminidase A and beta-hexosaminidase B. Both enzymes have two subunits. Beta-hexosaminidase A is made from one alpha subunit produced from the *HEXA* gene and one beta subunit (produced from the *HEXB* gene). However, beta-hexosaminidase B is only produced from two beta subunits encoded from the *HEXB* gene. Beta-hexosaminidase A and B, which are mainly found in lysosomes, break down sphingolipids, oligosaccharides and glycoproteins, and are critical in the central nervous system as they have very essential roles in breaking down the GM2 ganglioside in this system, preventing the accumulation of this product and its damage to the nervous system.^1–3^

According to the Human Gene Mutation Database (HGMD, http://www.hgmd.cf.ac.uk/ac/index.php), more than 182 disease-causing mutations of the *HEXA* gene have been identified in patients affected by Tay-Sachs disease (MIM #272800), including missense/nonsense, 96; splicing, 33; small deletions, 28; small insertions, 8; small indels, 3; and gross deletions, 1 (7.6 kb incl. promoter+ex. 1). Regarding the *HEXB* gene, to date, more than 107 pathogenic mutations in this gene have been reported in patents affected by Sandhoff disease (MIM #268800), including missense/nonsense, 39; splicing, 18; small deletion, 18; small insertion, 3; and gross deletion, 6. These mutations result in the accumulation of GM2 ganglioside in the central nervous system, damaging this systems and presenting the signs and symptoms identified in patients with the corresponding disorders. On the basis of the fully nonfunctional HEXA and HEXB proteins or reduced activity of these proteins resulting from mutations in their genes, these disorders are presented as a severe form found in infancy or a less severe type with a later onset. Tay-Sachs and Sandhoff diseases (which are clinically indistinguishable from each other) are autosomal recessive, progressive neurodegenerative disorders. Their classical infantile forms develop in infancy with developmental retardation, paralysis, dementia, blindness and death usually by 2 or 3 years of age.^1,2,4–6^ A study conducted by Smith *et al.*^3^ showed that the clinical findings in infantile-onset disease were generally as follow: hyperacusis with excessive startle response in 85% of individuals; increased myotatic reflexes in 85% of individuals (children with infantile-onset disease were not able to walk independently); a pattern of combined axial hypotonia with limb spasticity in 86% of individuals; visual deterioration in 89% of individuals; and seizures in 84% of individuals. Considering the importance of identifying genetic causes in our patients that are suggestive of Tay-Sachs and Sandhoff diseases, the aim of our study was to identify disease-causing mutations in these patients from the south of Iran and report their clinical findings.

## Materials and methods

Ten unrelated patients suspected of GM2 ganglioside diseases from the south of Iran were recruited in the current study. All patients gave informed consent before undergoing genetic analysis of the *HEXB* and *HEXA* genes based on the requirements of the ethics committee in Comprehensive Medical Genetics Center, Shiraz University of Medical Sciences. Three milliliters of whole-blood samples from patients was collected in EDTA tubes and stored at −20 °C until use. Genomic DNA was prepared from peripheral blood lymphocytes using a DNA extraction kit (Yekta Tajhiz, Tehran, Iran) according to the manufacturer’s instructions. The genomic DNA concentration was measured by a NanoDrop (ND1000, NanoDrop Technologies, Wilmington, DE, USA) and stored at –20 °C until use.

All of the exon and exon–intron boundaries of the *HEXA* and *HEXB* genes were amplified by the PCR primers given in Table 1. These primer pairs were designed and evaluated according to their reference genomic sequences in ENSEMBLE (https://www.ensembl.org) and the use of NCBI BLAST(https://blast.ncbi.nlm.nih.gov), ENSEMBLE BLAST/BLAT (https://www.ensembl.org), UCSC (BLAT and In Silico PCR, https://genome.ucsc.edu) and Allele ID 7.5 (a bioinformatics software that helps design primers and probes). The total volume of the PCR reaction was 50 μl, which contained 1 μl of the forward and reverse primers (20 pmol/μl), 3 μl of DNA template (50–200 ng), 25 μl of TEMPase Hot Start 2x Master Mix Blue (Ampicon, A290806) and 20 μl of dH2O. PCR was carried out according to the Amplicon TEMPase Hot Start program. Ten microliters of PCR amplicons were visualized on a 2% agarose gel containing SYBR Safe (Invitrogen, Paisley, Scotland, UK). Then, the PCR products were sequenced using Sanger sequencing, and their results were analyzed using NCBI BLAST and Codon Code Aligner software (CodonCode Corporation, Centerville, MA, USA).

In addition, to predict the cause of a new large deletion in the *HEXA* gene due to *Alu* elements, RepeatMasker (http://www.repeatmasker.org) was used with the RMBlast search engine (a version that was compatible with the NCBI Blast tool suite). The Repeat masker program screens DNA sequences of interspersed repeats and low complexity DNA sequences. Sequence comparisons in RepeatMasker are carried out using one of the different search engines, including nhmmer, cross_match, ABBlast/WUBlast, RMBlast and Decypher. RepeatMasker uses information from curated libraries of repeats, Dfam and Repbase. We investigated any *Alu* repeats (especially *Alu* repeats flanking deleted exons) in the whole genomic structure of *HEXA*, covering a total of 34,695 bp, starting 1.4 kb upstream of exon 1 to 1.4 kb downstream of exon 14. Moreover, to identify the breakpoint of the identified large deletion around exons 6 and 10 in our patient, we performed a second PCR around the predicted *Alu* elements using the primers given in Table 1 (the two forward primers upstream of *Alu*Jo and *Alu*Sx in intron 5, and the reverse primers downstream of *Alu*Jo in intron 10). The PCR products were then used for Sanger sequencing. In addition, a coding sequence translator (http://www.fr33.net/translator.php) was used to predict the possible effect of this deletion on the amino-acid sequence of the HEXA protein.

To predict the functional effects and conservation status of a newly identified missense mutation in another patient, we also used different bioinformatics tools, including MutationTaster, MutationAssesor, Polyphen, Grantham, PhastCons, GERP, CADD, Fathmm and SIFT. The T-coffee multiple sequence alignment program (http://www.ebi.ac.uk/Tools/msa/tcoffee/) was applied to compare the amino-acid sequence of *HEXA* proteins across different species to obtain information about the conservation of amino acids at the site of mutation.

## Results

**Figure 1:**
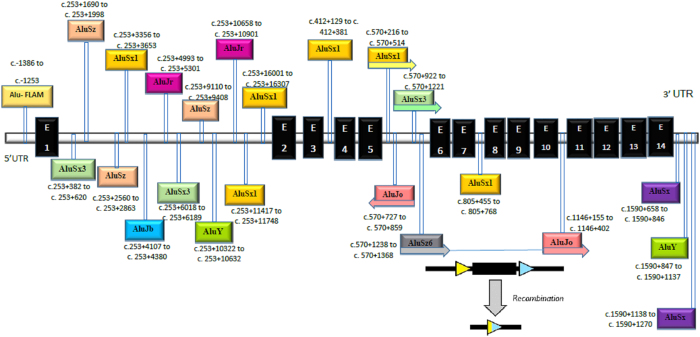
Different *Alu* elements across the *HEXA* gene. The most prevalent *Alu* element across this gene are the *Alu*S class, mainly *Alu*Sx. As seen, the possible mechanism for the deletion of exons 6–10 is recombination between *Alu*Sz6 in intron 5 and *Alu*Jo in intron 10.

**Figure 2:**
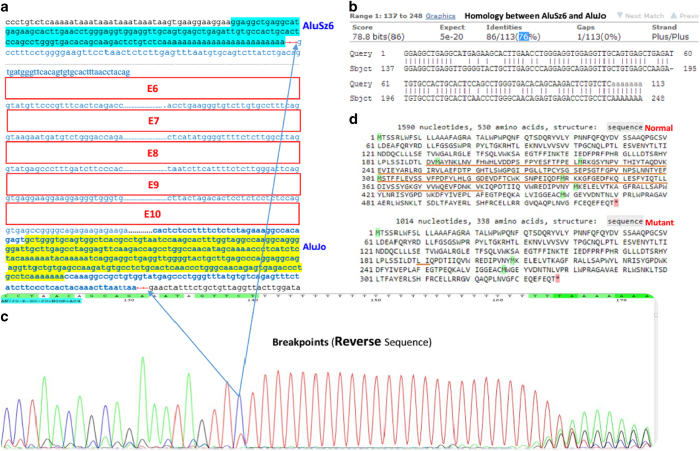
The breakpoint of the deletion and deleted region in the *HEXA* gene. (**a**) The red arrows show the breakpoint of the deletion (the region between these red arrows was completely deleted), blue arrows show the deleted region in the Sanger sequencing chromatogram and blue letters indicate the deleted nucleotides, including all of exons 6–10. Blue-highlighted nucleotides show the sequence of *Alu*Sz6, and yellow-highlighted nucleotides represent *Alu*Jo. (**b**) The homology between *Alu*Sz6 and *Alu*Jo is indicated as 76%, which is used for recombination between these two *Alu* elements. (**c**) Sequencing data confirmed that the breakpoint of the large deletion in *HEXA* was due to the *Alu* elements. (**d**) Normal and mutant HEXA proteins that resulted from the large deletion are compared, indicating the loss of 192 amino acids from the protein. In the normal sequence, the brown underline shows the deleted residues, and in the mutant sequence, the underline shows the breakpoints of the deletion and also the two joined amino acids after this deletion.

**Figure 3:**
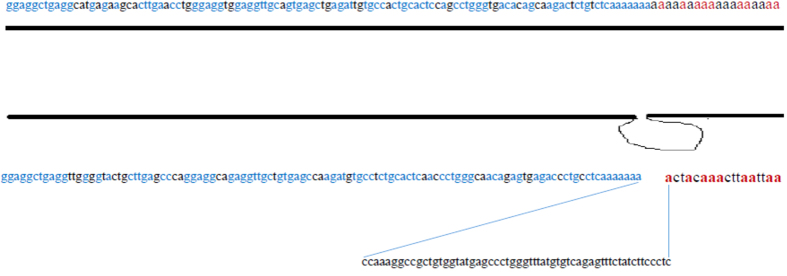
The similarity between *Alu*Sz6 and *Alu*Jo and its flanking sequence. There was high homology between these *Alu* elements, and the microhomology between 18 nucleotides of the *Alu*Sz6 and the sequence at the breakpoints helps to determine the occurrence of recombination between these sequences, leading to *Alu*-mediated deletion. Blue letters show homology between *Alu*Sz6 and *Alu*Jo. Red letters show microhomology at the breakpoints.

**Figure 4:**
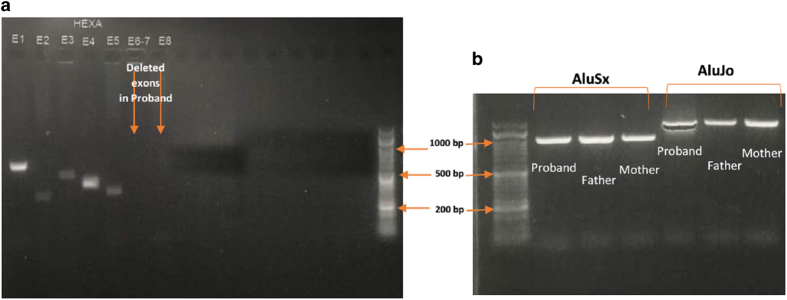
PCR products from patients with deletion of exons 6–10 in *HEXA*. (**a**) PCR for different exons of *HEXA* in patients for whom exons 6–10 were deleted as there were no PCR bands for them (here, only we show deletion of exons 6–8 from this deleted block). (**b**) PCR products using primers upstream of *Alu*Jo and *Alu*Sx in intron 5 and downstream of *Alu*Jo in intron 10. PCR revealed that the proband with the exon 6–10 deletion only had PCR bands produced from primers designed around the *Alu* elements (1,000 bp for *Alu*Sx-*Alu*Jo and 1,500 bp for *Alu*Jo-*Alu*Jo), revealing that the homozygous status due to the amplification of the reduced size of the exon 6–10 block resulted from this deletion. In our patient’s parents, not only were the bands amplified from the primers around the *Alu* elements detected but all of the exons of *HEXA* also produced PCR bands (here, only the PCR bands around the *Alu* elements are given for parents), indicating its heterozygous status. E, exon.

One known (NM_000520 (HEXA-201): c.346+1G>A, IVS2+1G>A)^7^ splice site and three novel *HEXA* mutations, including NM_000520 (HEXA-201): c.919G>A [p.E307K], the deletion of exons 6–10 in a homozygous state and NM_000520 (HEXA-201): c.622delG [p.D208Ifsx15], which are suspected as being homozygous, were identified. One variant of uncertain significance (NM_000521 (HEXB-201): c.619A>G [p.I207V])^8^ and one known mutation (NM_000521 (HEXB-201):c.1597C>T [p.R533C])^9^ in *HEXB* were found in a homozygous state (Table 2). From 10 patients, 5 cases only had the homozygous p.R533C mutation in *HEXB*, indicating its high frequency in our population. For the first time, a large homozygous deletion (deletion of exons 6–10) in *HEXA* was identified in our patient. Using the repeat masker tool, different *Alu* elements were found across the *HEXA* gene (Figure 1), including one *Alu* at the 5ʹ untranslated region, twelve in intron 1, one in intron 3, four in intron 5 flanking exon 6 (the first exon which was deleted in a block of five exon deletions), one in intron 7, one in intron 10 flanking exon 10 (the last exon which was deleted in the deleted block) and three *Alu*s at the 3ʹ untranslated region. The exact breakpoint of the deletion around exons 6 and 10 were determined using different PCR products flanking the *Alu* elements in their corresponding intron-flanked deleted exons. In addition, the consequence of this deletion was predicted to be an in-frame deletion of 192 amino acids of the HEXA protein, leading to its abnormal function (Figures 2a–d and 3). In patients with this deletion, PCR did not show any band for exons 6–10, but the patient had PCR bands around *Alu* elements in introns 5 and 10 (1,000 bp for *Alu*Sx-*Alu*Jo and 1,500 bp for *Alu*Jo-*Alu*Jo), revealing the homozygous status. In her parents, all of the exons produced PCR bands and bands around *Alu* elements, indicating its heterozygous status (Figure 4). Using PCR around *Alu* elements (*Alu*Jo and *Alu*Sx in intron 5, and *Alu*Jo in intron 10) followed by Sanger sequencing, the results revealed that the breakpoints of the large deletion occurred in *Alu*Sz6 (its length was 312 nucleotides, and its breakpoint was in nucleotide 307 of this *Alu*) in the intron 6 and 73 nucleotides downstream of *Alu*Jo in intron 10 (this deletion also contained the whole *Alu*Jo sequence; Figure 2a, c). The names of the *Alu* elements were determined from the results of RepeatMasker and were extracted from Dfam and Repbase.

**Figure 5:**
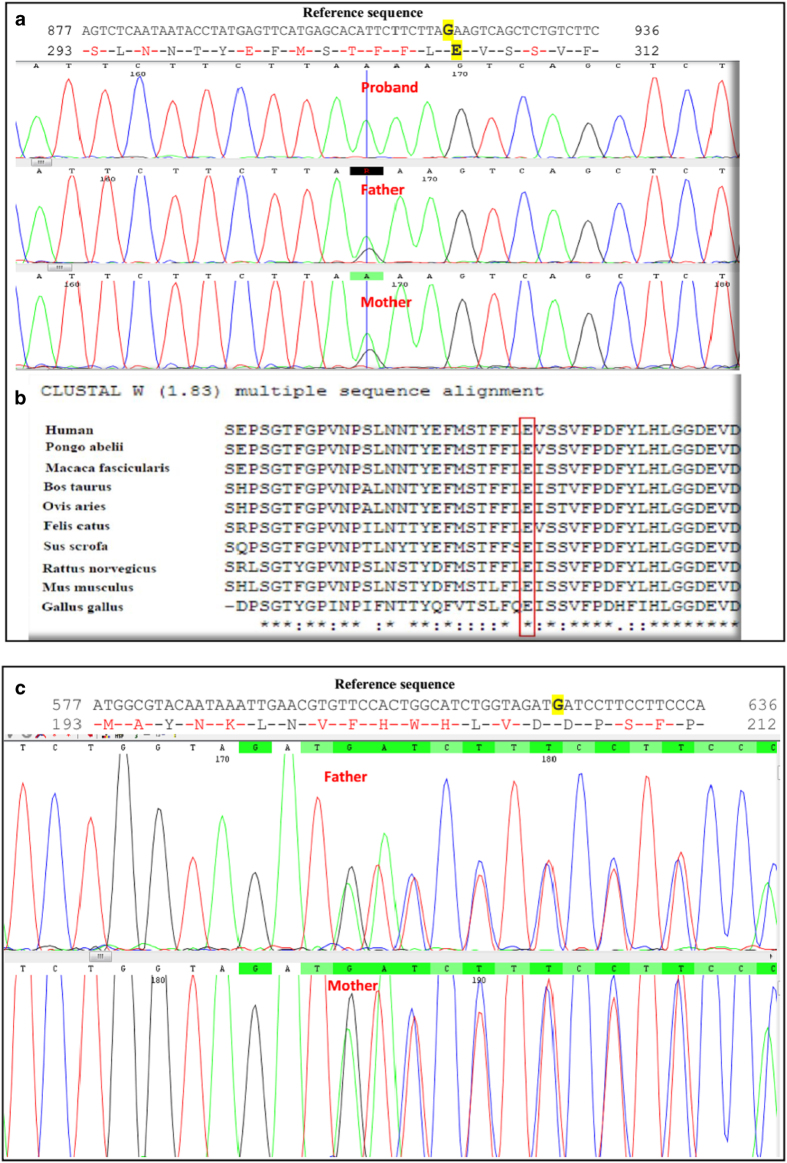
(**a**) Sanger data from the family with a novel c.919G>A (p.E307K) missense mutation. The reference sequence was obtained from the Ensembl database (https://www.ensembl.org/). Reference nucleotide and residues are highlighted in yellow. (**b**) Comparative alignment of the amino acids of HEXA across different species. The E307 residue is highly conserved during evolution. The conserved glutamic acid residue is shown in the rectangular box. Protein sequences were obtained from National Center for Biotechnology (NCBI). Symbols: (*)—identical amino acids; (:)—only similar amino acids. *Pongo abelii*: one of two species of orangutans; *Macaca fascicularis*: one species of macaque; *Bos taurus* (domestic cow): a species of oxen; *Ovis aries*: domestic sheep; *Felis catus*: domestic cat; *Sus scrofa*: wild boar or Eurasian wild pig; *Rattus norvegicus*: brown rat, also referred to as common rat, street rat and Norway rat; *Mus musculus*: house mouse; *Gallus gallus* (the red junglefowl): a type of domesticated fowl. (**c**) The Sanger chromatogram revealed a novel heterozygous c.622delG, p.D208Ifsx15 in both parents of the proband. Reference nucleotides are highlighted in yellow.

In addition to a novel large deletion, a new c.919G>A [p.E307K] missense mutation in *HEXA* was also identified as homozygous in one patient and was confirmed as being heterozygous using Sanger sequencing in her parents (Figure 5a). Moreover, different bioinformatics tools confirmed the pathogenicity of this mutation (Table 3). In addition, the T-coffee tool revealed that the glutamate residue at position 307 was highly conserved during evolution among different species (Figure 5b). Another new mutation, c.622delG [p.D208Ifsx15], was identified as being heterozygous in parents of the Case-MOE (Table 2 and Figure 5c), and since the death of patient was before the time of genetic testing, we only identified this deleterious mutation by performing Sanger sequencing of the whole *HEXA* and *HEXB* genes in both parents. Therefore, this case is suspected to be homozygous.

## Discussion

The current study revealed the structure and distribution of a large deletion mutation consisting of exons 6–10 of *HEXA* in Tay-Sachs disease. The deletion was 4.6 kb long and spanned from 576 bp upstream of exon 6 to 475 bp downstream of exon 10. As shown in Figure 1, the *Alu* elements are located across the normal *HEXA* gene, which mainly flank the deleted region, demonstrating the involvement of these elements in large deletions in *HEXA*.

**Alu** elements are short interspersed elements that are moderately repetitive sequences and have a copy number of more than 1 million copies across the human genome (~11% of human genome). **Alu** elements are considered to be one of the most successful mobile elements.^10,11^ They are ~300 nucleotides in length and have a dimeric structure. The 3ʹ-end of an **Alu** element has a poly A-like structure, which plays a key role in its amplification mechanism.^12–15^ There are different subfamilies of *Alu* sequences that share similar nucleotides in each group, and the most common *Alu* subfamily in the human genome is the *Alu* S class (the most common *Alu* is *Alu*Sx).^16^ *Alu* sequences are responsible for the instability of the genome, which resulted from intrachromosomal and interchromosomal recombination events between these sequences.^17,18^ Deletions due to *Alu* repeats have been identified in different previously reported genes, such as the low-density lipoprotein receptor,^19^ NACHT, leucine-rich repeat, PYD-containing 7^20^ and *HEXB* genes.^21^

It is worth noting that while most breakpoints of deletions occur in *Alu* elements, one of the deletion breakpoints in our study was observed 73 bp downstream of the *Alu*Jo element in intron 10 (*Alu*Jo and 73 bp after this element were deleted along with the deleted block). As shown in Figures 1 and 2, the deletion occurred due to recombination between *Alu*Sz6 and *Alu*Jo as the breakpoint occurred in the last nucleotides of *Alu*Sz6 and 73 bp downstream of *Alu*Jo. Although there is an *Alu*Jo in intron 6, its direction is opposite of *Alu*Jo located in intron 10 (Figure 1, the same direction is needed to cause a deletion when recombination between *Alu*/*Alu* occurs). In addition, the similarity between *Alu*Sz6 and *Alu*Jo is 76% (Figure 2b), which is suitable for recombination as *Alu*/*Alu* recombination occurs when *Alu* elements are completely identical (homologous). In addition, in most of the observed events, *Alu* has a mismatch (homeologous) at the start of recombination.^11,22–24^

An explanation of why the breakpoints in intron 10 occurred 73 bp downstream from *Alu*Jo is that that some microhomology was observed at this breakpoint, such as with nucleotide adenine (shown as red letters in Figure 3). This microhomology, along with the homology between *Alu*, makes it possible to observe these *Alu*/*Alu* deletions. It has been shown that these deletions are due to pathways such as microhomology-mediated end joining, which occurs when there are 5–25 nucleotides of microhomology between two *Alu* elements.^25,26^ Therefore, as shown in Figure 3, microhomology between two *Alu* elements can be one of the main causes of the large deletion in the *HEXA* gene.

The c.919G>A missense mutation in exon 8 of *HEXA* was a novel mutation and resulted in a substitution from glutamate, a negatively charged, polar amino acid (at position 307) to lysine, a positively charged, polar amino acid (p.E307K). This amino acid is predicted to be essential for the proper functions of this protein (Table 3) and is conserved in the evolution of different species, such as *Mus musculus*, *Gallus gallus*, *Ovis aries* and many others (Figure 5b). Moreover, Sanger sequencing of all of exons and exon/intron boundaries of the *HEXA* and *HEXB* genes in the proband only identified this mutation in exon 8 of *HEXA*. On the basis of our results, this new variation is a real mutation and is predicted to have damaging effects on the protein.

Regarding c.622delG and p.D208Ifsx15, it is obvious that this mutation causes a frameshift deletion that led to a premature translation termination. It is worth noting that one of the reported transcripts of *HEXA* (ENST00000569410.5, H3BTD4, http://www.ensembl.org), which lacks all of the amino acids after position 373, caused nonsense-mediated decay. Therefore, all of these amino acids are vital for the normal function of the HEXA protein. Since the only identified damaging variation across *HEXA* and *HEXB* in both parents of the patient was this deletion and all of the phenotypes of the patient overlapped with the clinical abnormalities found in Tay-Sachs disease, this frameshift mutation can be reported to be the genetic cause of Tay-Sachs disease in this family.

One previously reported variant of uncertain significance (c.619A>G [p.I207V]) that has a high allele frequency (0.15148 for G) in the main database, ExAC (http://exac.broadinstitute.org), was identified as homozygous in one of our patients (Case-NEGAH, Table 2). There are conflicting interpretations of this variant since some studies have reported that the c.619A>G variant converts the Ile to Val (in position 207) in a highly conserved region of the HEXB protein, which is considered to be associated with alterations in the catalytic activity and activator protein binding^8^. However, there are several reports that indicate that this variant is benign^27,28^ or likely benign.^29^ Studies conducted by Redonnet-Vernhet *et al.*^28^ showed that the healthy mother of the patient who was affected by Sandhoff’s disease due to a mutation of p.R505Q was homozygous for p.I207V, indicating that this variant did not cause Sandhoff’s disease. Therefore, we suggest that the c.619A>G variant identified in our patient cannot be the genetic cause of Sandhoff’s disease and other genes with a clinical diagnosis that overlapped this disease may be involved in this case, and other genetic tests are needed, such as whole-exome sequencing.

Current studies have shown that *HEXA* is an *Alu*-rich gene and is predisposed to disease-associated large deletions. We also found different mutations in the *HEXA* and *HEXB* genes and that the most common mutation in *HEXB* was c.1597C>T, which suggests that screening the *HEXB* gene for this prevalent mutation is a cost-effective test in individuals with suspicion of having Sandhoff disease. Such studies can help understand the different possible mechanisms of genetic changes in different diseases and help to investigate large deletions in *HEXA* and *HEXB* in individuals affected by Sandhoff and Tay-Sachs diseases who do not have any point mutations or small deletions and insertions. In addition, with identification of all of the *Alu* elements across *HEXA* in this study, other researchers can look for any possible deletions more easily in special cases of Sandhoff and Tay-Sachs diseases. Until now, no drugs have been used to treat Sandhoff and Tay-Sachs diseases, and a better understanding of their exact pathomechanism in the future can shed light on therapeutic approaches for these diseases using components involved in their biological processes.

## Publisher's Note

Springer Nature remains neutral with regard to jurisdictional claims in published maps and institutional affiliations.

## Figures and Tables

**Table 1:** All of the primer pairs used in the current study

*HEXA primer pairs*		*HEXB primer pairs*	
FE1	5ʹ- CGGTTATTTACTGCTCTACTGG-3ʹ	FE1	5ʹ- TGTTTGAGGTTGCTGTCTGG-3ʹ
RE1	5ʹ- GAAGTGGAGTGCCTGTGA-3ʹ	RE1	5ʹ- TCGAAACTGAGGACTTCTGC-3ʹ
FE2	5ʹ- TCCTCCCTTTCCTTTACC-3ʹ	FE2	5ʹ- ATCTCTAGTTGGACTTACAATG-3ʹ
RE2	5ʹ- CGAGCATCAGCAGTTTAG-3ʹ	RE2	5ʹ- CTCATCCATATAGTGACAGAAC-3ʹ
FE3	5ʹ- GACATCTCCTCATTGAAAGA-3ʹ	FE3	5ʹ- TGAAATGAGGAACACAGAAGAC-3ʹ
RE3	5ʹ- ACACCTGTAATCCCAGAAC-3ʹ	RE3	5ʹ- TCACAAAGCACCAACTGAA-3ʹ
FE4	5ʹ- AACCCTTACTCTGACATCTC-3ʹ	FE4-5	5ʹ- TTAGAGGATAGAGGTATAACACTA-3ʹ
RE4	5ʹ- CACTCACATCTCCTCTTCCA-3ʹ	RE4-5	5ʹ- AAACAGGAGGGTGATTCTC-3ʹ
FE5	5ʹ- TGAGAACAGTCACAGATTG-3ʹ	FE6	5ʹ- AAGCAGACATATTGGAAGCA-3ʹ
RE5	5ʹ- ACACCCAGTCCATACATTC-3ʹ	RE6	5ʹ- AGCCTACATACCTAACATTGG-3ʹ
FE6-7	5ʹ- ATGGGAAGGTTTGATAGAC-3ʹ	FE7	5ʹ- TCCTTTGAGTATGTACGACTTAG-3ʹ
RE6-7	5ʹ- TCACTCTGAGCATAACAAG-3ʹ	RE7	5ʹ- CAGTGAGCCGAGATTGTAC-3ʹ
FE8	5ʹ- GTGTGACTCGTGTCCTTAC-3ʹ	FE8	5ʹ- AAGAGACAGGATTCAGGA-3ʹ
RE8	5ʹ- AAGACCTGAGCAATGTGAG-3ʹ	RE8	5ʹ- AGTACAGTGGCATGATCTCAG-3ʹ
FE9-10	5ʹ- CCACCTGCTTCACATAACT-3ʹ	FE9	5ʹ- AGGTGGTAAGGTAAAGAAAGC-3ʹ
R9-10	5ʹ- GAGAGTGCTCCGACCATTA-3ʹ	RE9	5ʹ- AAGCAAGCAGTGGGTATTG-3ʹ
FE11-12-13	5ʹ- CATGTTGCCTGTGTATGAAT-3ʹ	FE10-11	5ʹ- CTATGTTAGGTTCTGTATCAAG-3ʹ
RE11-12-13	5ʹ- AAGCCTCTGTAAGTGTTAGC-3ʹ	RE10-11	5ʹ- ACAATGGTTGCTTCACTTAC-3ʹ
FE14New	5ʹ- ACAGTGACAGTTTGAGAGTC-3ʹ	FE12-14	5ʹ- ATTGCCTCTGTGTATAAG-3ʹ
RE14New	5ʹ- TGCCTATTAATTCCAGTTACT-3ʹ	RE12-14	5ʹ- TAATGAGAAATGTGCTTTG-3ʹ
FHEXA-*Alu*Sx	5ʹ- TTCTAGGGTGATGGAGCAAC-3ʹ		
FHEXA-*Alu*Jo	5ʹ- TGAGCTGCTCCAAGACTCAA-3ʹ		
RHEXA-*Alu*Jo	5ʹ- AAGGTTCCTGAAGAGTCCTTG-3ʹ		

**Table 2:** Identified mutations in *HEXA* and *HEXB*, and the clinical and laboratory findings in this study

*Patients*	*Mutation*	*Gene*	*Exon*	*Novel or known*	*Laboratory findings*	*Clinical findings*
Case-AMA	Homo IVS2+1G>A	*HEXA*		Known	NA	Onset: 1 year Death: 3.8 years Psychomotor degeneration, mild vision deficiency, recurrent seizures, paralysis
Case-MOE	Suspected Homo c.622delG in her both parent	*HEXA*	6	Novel	NA	Onset: 6 months Death: 4.5 years Seizures (sometimes), mild vision deficiency, psychomotor degeneration, paralysis
Case-DAN	Homo exon 6–10 deletion (new)	*HEXA*		Novel	HexA: 0.00 nmol/ml/min (normal: 0.96–1.78) HexAB: 51.66 nmol/ml/min (normal: 18.59–31.33) HexB: 54.51 nmol/ml/min (normal: 5.76–15.77)	Onset: 5 months Death: 2.6 years Psychomotor degeneration, mild vision deficiency, seizures (sometimes), paralysis
Case-RAM	Homo c.919G>A, p.E307K	*HEXA*		Novel	NA	Onset: 8 months Death: 2.1 years Recurrent seizures, vision deficiency, psychomotor degeneration, paralysis
Case-NEGAH	Homo c.619A>G, p.I207V	*HEXB*		VOUS	NA	Onset: 7.5 months Death: 2.5 years Seizures (rarely), vision deficiency, psychomotor degeneration, paralysis
Case-MOHR	Homo c.1597C>T, p.R533C	*HEXB*		Known	HexAB: 0.2 mU/ml (normal: 16.7–40.7) HexA: 0.02 mU/ml (normal: 0.47–2.60) HexB: 0.02 mU/ml (normal: 3.94–14)	Onset: 8 months Death: 2.5 years Psychomotor degeneration, vision deficiency, seizures (sometimes), paralysis
Case-HANIE	Homo c.1597C>T, p.R533C	*HEXB*		Known	HexAB: 0.8 mU/ml (normal: 16.7–40.7) HexA: 0.11 mU/ml (normal: 0.47–2.60) HexB: 0.06 mU/ml (normal: 3.94–14)	Case-YAS Onset: 9 months Death: 2 years Psychomotor degeneration, vision deficiency, recurrent seizures, paralysis
Case-YAS	Homo c.1597C>T, p.R533C	*HEXB*		Known	HexAB: 0.0 mU/ml (normal: 16.7–40.7) HexA: 0.06 mU/ml (normal: 0.47–2.60) HexB: 0.02 mU/ml (normal: 3.94–14)	Onset: 1.6 year Death: 5 years Psychomotor degeneration, vision deficiency, recurrent seizures, hypotonia, paralysis
Case-TALP	Homo c.1597C>T, p.R533C	*HEXB*		Known	NA	Onset: 1.6 year Death: 5 years Psychomotor degeneration, vision deficiency, seizures, paralysis
Case-SADA	Homo c.1597C>T, p.R533C	*HEXB*		Known	NA	Onset: 6 months Death: 4.5 years Psychomotor degeneration, vision deficiency, seizures (at the late stage of disease), hypotonia, poor head control, paralysis

Abbreviations: HexA: beta-hexosaminidase A, HexB: beta-hexosaminidase B, HexAB: beta-hexosaminidase A and B; Homo, homozygous; NA, not applicable; VOUS, variant of uncertain significance.

**Table 3:** Bioinformatics analysis of the c.919G>A[p.E307K] in *HEAX* with their corresponding damage scores

*Bioinformatics tool*	*Score or effect*	*Bioinformatics tool*	*Score or effect*
PolyPhen	1	GranthamScore	56
ScoreCADD	35	ChimpAllele	C
AsianHapMapFreq	NA	DbSNPValidation	NA
SIFT:DAMAGING	0	Fathmm	DAMAGING, −4.74
ConsScoreGERP	5.6	EuropeanHapMapFreq	NA
Mutation Assessor	4.425	MutationTaster	Disease-causing
ScorePhastCons	0.99	AfricanHapMapFreq	NA

Abbreviation: N/A, not applicable.
